# The IL-17A rs2275913 single nucleotide polymorphism is associated with protection to tuberculosis but related to higher disease severity in Argentina

**DOI:** 10.1038/srep40666

**Published:** 2017-01-18

**Authors:** A. Rolandelli, R. E. Hernández Del Pino, J. M. Pellegrini, N. L. Tateosian, N. O. Amiano, S. de la Barrera, N. Casco, M. Gutiérrez, D. J. Palmero, V. E. García

**Affiliations:** 1 Departamento de Química Biológica. Facultad de Ciencias Exactas y Naturales. UBA, Intendente Güiraldes 2160, Pabellón II, 4°piso, Ciudad Universitaria (C1428EGA), Buenos Aires, Argentina; 2 Instituto de Química Biológica, Facultad de Ciencias Exactas y Naturales (IQUIBICEN), UBA (Universidad de Buenos Aires)-CONICET, Intendente Güiraldes 2160, Pabellón II, 4°piso, Ciudad Universitaria (C1428EGA), Buenos Aires, Argentina; 3 Centro de Investigaciones y Transferencia del Noroeste de Buenos Aires (CIT NOBA), CONICET. Newbery 261, Junín (6000), Buenos Aires, Argentina; 4 Instituto de Medicina Experimental-CONICET-Academia Nacional de Medicina. Pacheco de Melo 3081 (CP1425), Buenos Aires, Argentina; 5 División Tisioneumonología Hospital F.J. Muñiz, Uspallata 2272, (C1282AEN) Buenos Aires, Argentina; 6 Sección Bacteriología de la Tuberculosis, Hospital General de Agudos “Dr. E. Tornu”, Combatientes de Malvinas 3002, (C1427ARN) Buenos Aires, Argentina

## Abstract

*Mycobacterium tuberculosis (Mtb*) causes nearly 10 millions of new tuberculosis disease cases annually. However, most individuals exposed to *Mtb* do not develop tuberculosis, suggesting the influence of a human genetic component. Here, we investigated the association of the rs2275913 SNP (G → A) from IL-17A and tuberculosis in Argentina by a case-control study. Furthermore, we evaluated *in vitro* the functional relevance of this SNP during the immune response of the host against *Mtb* and analyzed its impact on clinical parameters of the disease. We found an association between the AA genotype and tuberculosis resistance. Additionally, within the healthy donors population, AA cells stimulated with a *Mtb* lysate (*Mtb*-Ag) produced the highest amounts of IL-17A and IFN-γ, which further support the genetic evidence found. In contrast, within the tuberculosis patients population, AA *Mtb*-Ag stimulated cells showed the lowest immunological parameters and we evidenced an association between the AA genotype and clinical parameters of disease severity, such as severe radiological lesions and higher bacilli burden in sputum. Overall, our findings demonstrated that the AA genotype from the IL-17A rs2275913 SNP is positively associated with protection to active tuberculosis but related to higher disease severity in the Argentinean population.

*Mycobacterium tuberculosis (Mtb*) infects 2 billon persons around the world, causing nearly 10.4 million of new active tuberculosis cases and 1.8 million of deaths annually^1^. In Argentina, last reports estimated 8933 of new tuberculosis cases and 570 deaths in 2013, clearly demonstrating the importance of this disease^2^. However, most individuals exposed to *Mtb* do not develop active disease, suggesting that both host genetic and environmental factors might influence the susceptibility to tuberculosis^3^. Actually, family-based association studies in leprosy and tuberculosis evidenced the great influence of genes in the susceptibility to infectious diseases^4^. Efficient activation of a cellular immune response is crucial to establish a protective immune response against intracellular pathogens like *Mtb*, where cytokines play a crucial role in the process^5^,^6^. Thus, it is expected that some genetic variants of main cytokines that operate during host-pathogen interaction would be associated with a higher resistance or susceptibility to *Mtb* infection. In fact, inherited defects of the IL-12/IFN-γ pathway are related to Mendelian Susceptibility to Mycobacterial Disease, a disorder characterized by disseminated mycobacterial infections, denoting the importance of the IL-12/IFN-γ pathway in these infections, as well as the relevance of the host genetic background^7^.

Single nucleotide polymorphisms (SNPs) are mostly biallelic point mutations, present within a population in a frequency higher than 1%^4^. SNPs are also believed to be the main source of variability among humans, especially when they influence gene expression or function depending on their location in the DNA sequence. Moreover, since SNPs are relatively easy to be detected, they are considered as one of the best biological markers in association or case-control studies. Therefore, a large number of SNPs in cytokine loci have been described and studied in complex illnesses like infectious and autoimmune diseases and cancer^4^. Particularly in tuberculosis, several SNPs have been reported as possible causes of disease resistance/susceptibility. Among them, there were described variations in cytokine genes such as IFN-γ, TNF-α, IL-1, IL-6, IL-10, IL-12 and IL-17^8^,^9^,^10^,^11^,^12^,^13^. However, only one study in the Argentinean population has reported the association between an IL-12 SNP and tuberculosis^14^. Thus, it is important to investigate the role of potential genetic variations in molecules of the immune system that participate in the development of the disease in Argentina.

IL-17A is a key cytokine in host-pathogen interaction during *Mtb* infection. Accordingly, several reports indicated that CD4^+^ T cells producing IL-17A and IL-22 contribute to the adaptive immune response against *Mtb* in individuals exposed to the pathogen and in tuberculosis patients (TB)^15^,^16^. Moreover, although IL-17A is rapidly induced by γδ T cells during infection^17^, IL-17A secretion by CD4^+^ T lymphocytes is required to eliminate primary infection and for the establishment of an effective memory response^18^,^19^,^20^,^21^. Previously, we demonstrated that peripheral blood mononuclear cells (PBMC) from TB stimulated with a lysate of *Mtb (Mtb*-Ag) secreted lower levels of IFN-γ and higher amounts of IL-17A as compared to healthy donors (HD)^22^. Furthermore, we showed that addition of recombinant IL-17A to PBMC inhibited IFN-γ production against *Mtb-*Ag^23^. We also demonstrated that CD4^+^ IFN-γ^+^ IL-17A^+^ lymphocytes are the main source of IL-17A produced by PBMC from TB in direct correlation with disease severity^22^. Other authors have reported that infection with antibiotic resistant *Mtb* strains induced higher numbers of T cells secreting IL-17A as compared to infection with susceptible *Mtb* strains, and this expression is associated with high antigen load^24^.

In particular, the rs2275913 SNP, produced by a substitution of the G by an A nucleotide base in the IL-17A gene promoter, is significantly associated with a vast number of diseases^9^,^25^,^26^,^27^,^28^,^29^. It has been reported that allelic variants of the rs2275913 SNP differentially bind the transcription factor NFAT, leading to differences in IL-17A secretion^30^. Three previous reports demonstrated an association between the rs2275913 SNP and tuberculosis in populations from Spain, Brazil and China^9^,^10^,^13^. Therefore, the aim of this study was to investigate the potential association of the IL-17A rs2275913 SNP and tuberculosis in Argentina. Moreover, we also evaluated the functional relevance of this SNP during the immune response of the host against *Mtb*, even by analyzing its association with clinical parameters of the disease.

## Results

### Demographic characteristics of the population studied

In order to investigate the association between IL-17A rs2275913 SNP and tuberculosis in Argentina, 185 TB and 207 HD were recruited between 2013 and 2016. Demographic characteristics of both populations are shown in Table 1. We did not observed differences regarding ethnic origin between individuals of both populations (*P* > 0.05), nor in age distribution (*P* > 0.05). Nevertheless, we observed differences in the percentages of recruited individuals of each sex in both populations (*P* < 0.001), but we did not find differences between the genotype distributions by sex within each population (Supplementary Table S1). Therefore, differences between the percentages of individuals from both sexes would not affect the genotype distribution analyzed in each population.

### Genotypic and allelic frequencies of the IL-17A rs2275913 SNP in HD and TB populations

**Figure 1:**
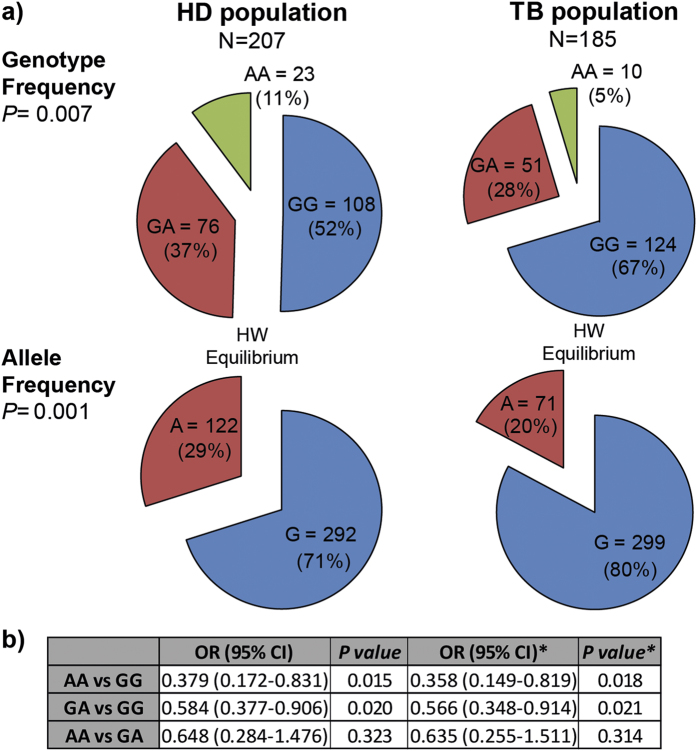
Genotypic and allelic frequencies of the IL-17A rs2275913 SNP in HD and TB populations in Argentina. **(a)** Pie chart representing the genotypic and allelic distribution of the rs2275913 SNP in both populations. The number of individuals of each population and the frequencies (in parentheses) are detailed. *P* values were calculated by the Chi-Square test of homogeneity. Both populations are in Hardy-Weinberg (HW) equilibrium. **(b)** Odds ratio calculation was used to quantify the association between tuberculosis and the different genotypes. *P* values were calculated by the Fisher test. *Values from a logistic regression model adjusted by sex, ethnicity and age. HD: healthy donors; TB: tuberculosis patients; H.W.: Hardy-Weinberg.

Genotyping of the rs2275913 SNP in HD and TB populations was performed by the ARMS-PCR technique as described in Material and Methods section (Supplementary Figure S1). Figure 1a shows the genotypic and allelic frequencies distribution observed in both populations. Importantly, we found that both populations were in Hardy-Weinberg (HW) equilibrium. Chi-Square test of homogeneity showed that HD and TB populations were significantly different regarding the genotypic and allelic frequencies (*P* < 0.01). In fact, both the A allele and the AA genotype were found in a lower proportion in TB population. Odds ratios were calculated to estimate the level of association between the rs2275913 genotypes and tuberculosis disease (Fig. 1b)^31^. When we compared the AA genotype against GG, the odd ratio value was 0.379 (*P* < 0.05); and by comparing individuals with the GA against GG genotypes, we observed an odd ratio of 0.584 (*P* < 0.05). Taken together, these data demonstrate an association between the A allele and the AA genotype with a reduced frequency of individuals suffering from tuberculosis, suggesting a potential relationship between the AA genotype of the rs2275913 SNP and protection against this disease in Argentina.

### IL-17A and IFN-γ plasma levels from HD and TB carrying the rs2275913 SNP variants

**Figure 2:**
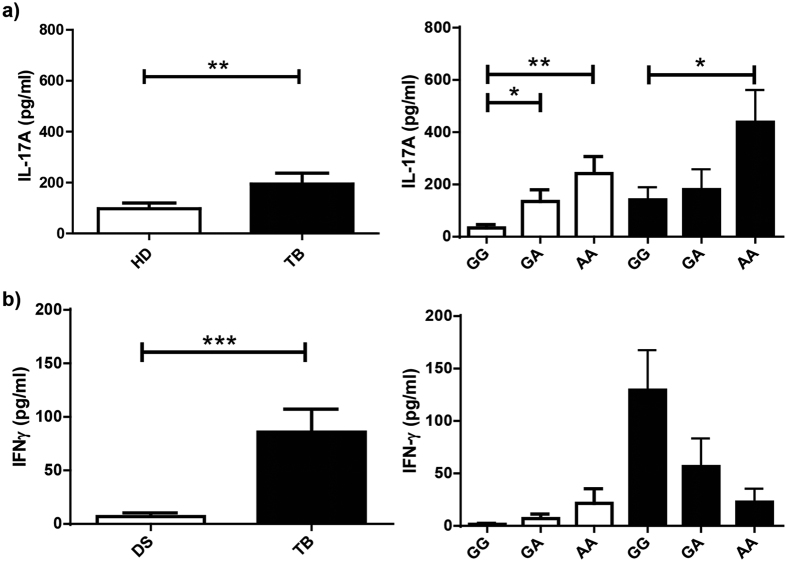
IL-17A and IFN-γ levels in plasma from HD and TB carrying the genotypic variants of the rs2275913 SNP. Plasma samples from healthy donors (HD, white bars, n = 20) and tuberculosis patients (TB, black bars, n = 23) were obtained, and IL-17A **(a)** and IFN-γ **(b)** levels were measured by ELISA and classified by genotypes. Bars represent the Mean ± SEM. P values were calculated by the Mann–Whitney U test (**a** and **b**, left panel) or the Kruskal-Wallis (ANOVA) test (**a** and **b**, right panel) for unpaired and non-parametric samples. *P < 0.05; **P < 0.01; ***P < 0.001.

To deeply investigate the hypothesis relating to the AA genotype of the rs2275913 SNP with protection against tuberculosis disease, we next analyzed plasma levels of IFN-γ and IL-17A, two crucial cytokines that participate in the immune response against *Mtb*^7^,^22^,^23^,^32^. Then, we determined by ELISA the levels of these two cytokines among individuals carrying the different genotypes of the rs2275913 SNP (Fig. 2). As previously described^9^, we observed significantly higher plasma levels of IL-17A in the TB population as compared to the HD population (*P* < 0.01). Importantly, in both populations, AA individuals displayed significantly higher levels of IL-17A as compared to GG subjects (Fig. 2a, *P* < 0.05). Besides, in line with other report^33^, we detected significantly higher plasma levels of IFN-γ in TB as compared to HD (Fig. 2b). Interestingly, within the TB population, a marked trend to display the lowest plasma levels of IFN-γ was observed in AA carriers (*P* > 0.05), in clear contrast to HD, where the opposite trend was observed (Fig. 2b).

### IL-17A and IFN-γ production by PBMC from HD and TB carrying the rs2275913 SNP variants

**Figure 3:**
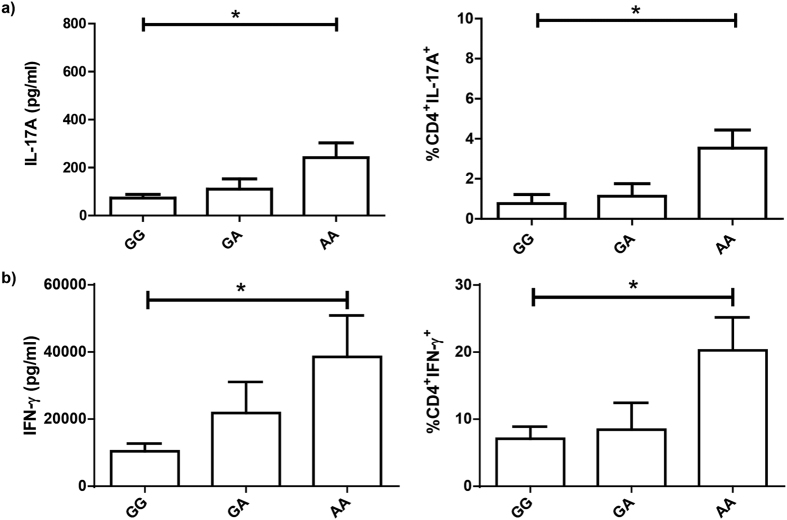
Production of IL-17A and IFN-γ by PBMC from HD carrying the genotypic variants of the rs2275913 SNP. PBMC from healthy donors (HD, n = 19) carrying the different genotypes of the rs2275913 SNP were stimulated for five days with *Mtb*-Ag, and IL-17A **(a)** and IFN-γ **(b)** production were determined by ELISA and Flow Cytometry. The percentages (right panel) represent an increase in the number of cytokine-positive CD4^+^ T cells in response to *Mtb-*Ag stimulation. IL-17A and IFN-γ expression was determined gating on lymphocytes by light scatter first, and then gating on CD4^+^ T cells. Bars represent the Mean ± SEM. *P* values were calculated by the Kruskal-Wallis (ANOVA) test for unpaired and non-parametric samples. *P < 0.05.

In order to get further evidences that support the protective role of the AA genotype in developing tuberculosis, we next stimulated PBMC from HD carrying the different genotypes of the rs2275913 SNP with *Mtb*-Ag, then IFN-γ and IL-17A production were determined by ELISA and Flow Cytometry. Figure 3 shows that AA HD displayed the highest IL-17A (*P* < 0.05) and IFN-γ (*P* < 0.05) levels in supernatants after five days of stimulation, as compared to the other genotypes. In line with these results, AA HD also displayed the greatest percentage of CD4^+^ IL-17A^+^ (*P* < 0.05) and CD4^+^ IFN-γ^+^ (*P* < 0.05) lymphocytes as compared to GG HD subjects. These data are in agreement with our hypothesis, suggesting that AA individuals present the lowest susceptibility to tuberculosis disease probably because they generate an effective immune response against the bacteria, producing higher levels of IL-17A and IFN-γ, which may contribute to eliminate the pathogen at a first contact^16^,^19^,^22^,^34^,^35^,^36^,^37^,^38^.

**Figure 4:**
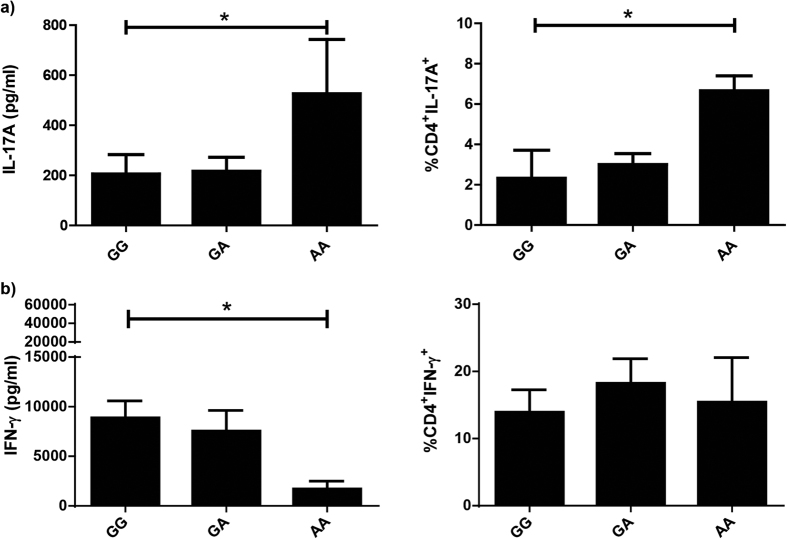
Production of IL-17A and IFN-γ by PBMC from TB carrying the genotypic variants of the rs2275913 SNP. PBMC from tuberculosis patients (TB, n = 19) carrying the different genotypes of the rs2275913 SNP were stimulated for five days with *Mtb*-Ag lysate, and IL-17A **(a)** and IFN-γ **(b)** production were determined by ELISA and Flow Cytometry. The percentages (right panel) represent an increase in the number of cytokine-positive CD4^+^ T cells in response to *Mtb*-Ag stimulation. IL-17A and IFN-γ expression was determined gating on lymphocytes by light scatter first, and then gating on CD4^+^ T cells. Bars represent the Mean ± SEM. *P* values were calculated by the Kruskal-Wallis (ANOVA) test for unpaired and non-parametric samples. *P < 0.05.

The fact that the AA genotype is the most protective one does not imply that individuals carrying this genotype display full immunity to tuberculosis. Therefore, if the subjects get tuberculosis disease, and considering they carry a genotype that induce their cells to produce higher levels of IL-17A^30^, that may expose these individuals to a worse disease outcome. For that reason, we next evaluated the production of IL-17A and IFN-γ in response to *Mtb*-Ag by PBMC from TB carrying different genotypes of the rs2275913 SNP. Interestingly, similar to the results observed in HD, the highest levels of IL-17A (*P* < 0.05) and the highest percentages of CD4^+^ IL-17A^+^ cells (*P* < 0.05) were observed in AA TB (Fig. 4a). Moreover, AA TB secreted levels of IL-17A that doubled those produced by AA HD (Fig. 3a). In contrast to our findings in HD (Fig. 3b), AA TB secreted the lowest levels of IFN-γ against *Mtb*-Ag as compared to TB carrying the other genotypes (*P* < 0.05, Fig. 4b). However, no differences in the percentages of IFN-γ secreting cells were detected among the three genotypes (Fig. 4b). These findings are in line with our previous results demonstrating that *Mtb*-Ag stimulation of PBMC from TB produced lower IFN-γ levels but higher IL-17A amounts in comparison to HD^22^,^39^. Moreover, we have also shown that high amounts of recombinant IL-17A in the cell culture inhibited the production of IFN-γ against the pathogen by PBMC^23^, according to our present data observed in AA TB, where we detected the lowest levels of IFN-γ in the presence of the highest levels of IL-17A (Fig. 4).

### Association between immunological and clinical parameters and the rs2275913 SNP variants during active tuberculosis

**Figure 5:**
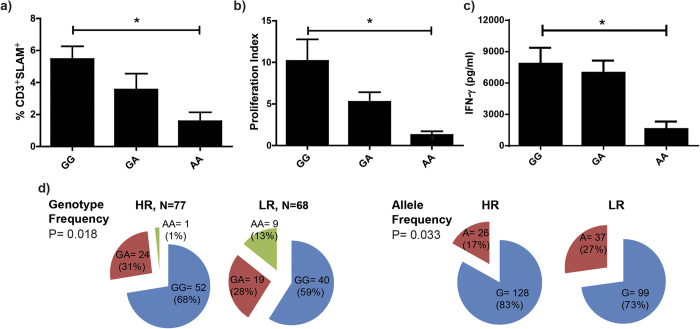
Association between immunological parameters and the rs2275913 SNP genotypic variants during active tuberculosis. **(a,b)** PBMC from tuberculosis patients (TB, n = 86) carrying the different genotypes of the rs2275913 SNP were stimulated for five days with *Mtb*-Ag. Then, **(a)** the expression of SLAM in CD3^+^ T cells was determinated by Flow Cytometry, and **(b)** the proliferation index (cpm after stimulation/cpm after unstimulation) was evaluated by [^3^H] thymidine incorporation. SLAM expression was determined gating on lymphocytes by light scatter first, and then gating on CD3^+^ T cells. **(c)** PBMC from TB (n = 86) carrying the different genotypes of the rs2275913 SNP were stimulated for two days with *Mtb*-Ag and IFN-γ production was determined by ELISA. Bars represent the Mean ± SEM. *P* values were calculated by the Kruskal-Wallis (ANOVA) test for unpaired and non-parametric samples. *P < 0.05. **(d)** Pie chart representing the genotypic and allelic distribution of the rs2275913 SNP in TB (n = 145) classified as High and Low responder (HR and LR, respectively) individuals according to their immune response. The number of individuals of each group and the frequencies (in parentheses) are detailed. *P* values were calculated by the Chi-Square test of homogeneity.

Our previous work evidenced that higher levels of IL-17A were directly associated with tuberculosis severity^22^. Therefore, considering that TB carrying the AA genotype secreted the lowest levels of IFN-γ and the highest IL-17A amounts against *Mtb*-Ag, we investigated whether AA TB might be the most affected by the disease. Thus, we initially evaluated the immunological parameters that we used to classify TB as high and low responder (HR and LR) individuals according to their immune response to *Mtb*-Ag *in vitro*^39^. Figure 5a–c shows the results for SLAM expression in CD3^+^ T cells, proliferation index and IFN-γ production by PBMC from GG, GA and AA TB. We observed that AA TB displayed significantly lower levels of SLAM expression (*P* < 0.05) and the lowest proliferation index (*P* < 0.05) after five days of stimulation, as compared to GG TB. Moreover, we detected the lowest IFN-γ secretion (*P* < 0.05) after two days of stimulation in AA TB as compared to the levels detected in TB carrying the other genotypes (Fig. 5). Furthermore, by evaluating the allelic and genotypic distribution in HR and LR TB we detected that the A allele and the AA genotype were overrepresented in LR patients (Fig. 5d,e, *P* < 0.05). Thereby, through immunological *in vitro* studies these results demonstrate that AA TB displayed the weakest responses against *Mtb*-Ag, and therefore they would be severely affected by the disease.

We have previously demonstrated that immunological features paralleled common clinical parameters analyzed in TB in Argentina: HR patients had significant higher percentages of total lymphocytes compared with LR patients; HR patients exhibited higher purified protein derivative diameters than LR patients; and LR individuals had severe pulmonary lesions, a striking loss of weight, and had been ill longer than HR individuals^39^,^40^. Therefore, considering our data on the association between the AA genotype and the immunological features in TB, we next analyzed the potential association between the AA genotype and clinical parameters usually studied in tuberculosis. As can be observed in Table 2, we did not find significant differences in leukocyte, lymphocyte, monocyte or neutrophil numbers in peripheral blood of TB carrying the different genotypes of the rs2275913 SNP. We neither detected differences in the time of disease progression. However, most of TB carrying the AA genotype (70%) displayed the highest bacilli burden in sputum in contrast to 70% of GG TB that displayed none or very few bacilli in sputum. Furthermore, radiological lesions indicated that 60% of AA TB displayed severe pulmonary conditions (bilateral disease with massive affectation and multiple cavities), in contrast to 57% of TB carrying the GG genotype that showed the mildest lung lesions. These findings demonstrate that TB carrying the AA genotype of the IL-17A rs2275913 SNP exhibit weak cell-mediated immunity against *Mtb*-Ag, in direct association with critical clinical parameters, indicating disease severity.

Overall, by comparing the HD and the TB populations, we found genetic evidences of an association between the IL-17A rs2275913 AA genotype and resistance to tuberculosis disease in Argentina, which was further supported by *in vitro* analyses. Additionally, by focusing only on the TB population, we detected a genetic evidence of an association between the AA genotype and tuberculosis severity, which was also supported by immunological and clinical parameters.

## Discussion

The rs2275913 SNP is the nucleotide variant of the IL17A gene associated with a vast number of pathologies^9^,^25^,^26^,^27^,^28^,^29^. By evaluating the distribution of the rs2275913 SNP in HD and TB population in Argentina we found significant differences in both allelic and genotypic frequencies. The AA genotype and the A allele showed the lowest frequencies in the TB as compared to HD population, suggesting an association of the rs2275913 SNP A variant with resistance to tuberculosis disease in Argentina. Interestingly, more than 80% of individuals from the HD population (168 out of 207) were subjects exposed to *Mtb* (tuberculosis contacts) but not infected with the pathogen (QFT-GIT negative), reinforcing that these individuals would be effectively resistant to *Mtb* infection. Remarkably, HD and TB populations showed HW equilibrium and no age or ethnic differences. Besides, although both populations displayed different sex proportions, those disparities would not have any impact in the frequency distribution of the rs2275913 SNP. Therefore, we conclude that none of the analyzed factors would influence in the allelic or genotypic frequencies distribution of the rs2275913 SNP in the populations under study.

In order to find an association with resistance/susceptibility to tuberculosis, several studies investigated the rs2275913 SNP in ethnically different populations^9^,^10^,^13^,^41^,^42^. In a Croatian^41^ and a Chinese^42^ populations, no association between the SNP and tuberculosis were detected. A possible cause of the discrepancies between those previous results and our present data might be related to ethnic differences. Nevertheless, another study in China demonstrated a relationship between the G allele and the GG genotype with resistance to tuberculosis disease^13^. However, the TB population studied did not show HW equilibrium. In contrast, both in a population from North Spain^9^ and another one at the South of Brazil^10^, populations in which the HW equilibrium condition was fulfilled, the A allele and the AA genotype displayed the lowest frequencies in the TB population, in agreement with our present findings. Thus, our data are consistent with previous reports, further supporting the idea of a higher resistance to tuberculosis disease in AA individuals and highlighting the important function of IL-17A in immunity against *Mtb*.

It has been demonstrated that the rs2275913 SNP is a functional polymorphism that modifies the binding of the transcriptional factor NFAT to the IL-17A promoter. The A variant allows a stronger binding of NFAT, leading to a higher transcription and synthesis of the IL-17A protein^30^. Accordingly, we observed elevated levels of IL-17A in subjects carrying the AA genotype as compared to individuals that carry the GG genotype, both in plasma and in supernatants of *in vitro* stimulated PBMC, and independently of the population analyzed. Thus, NFAT might be involved in this differential synthesis of IL-17A, given that this transcription factor promotes the transcription of genes during T lymphocyte activation and was detected in cells stimulated with *Mtb* antigens^30^,^43^,^44^. However, further studies are required to confirm this hypothesis.

Several reports have demonstrated the protective role of IL-17A during the early stages of *Mtb* infection, contributing to the recruitment of neutrophils and IFN-γ secreting cells to the site of infection to establish an effective memory response^18^,^19^. Interestingly, we observed that, within the HD population, individuals that carry the AA genotype displayed the highest levels of IL-17A in plasma, and produced the highest levels of this cytokine and showed the highest numbers of T lymphocytes secreting IL-17A upon stimulation of their PBMC with *Mtb*-Ag. Moreover, these subjects showed the highest levels of IFN-γ in plasma, a key cytokine in the defense against mycobacteria^7^,^32^,^34^, although no significant differences were observed. Besides, PBMC from AA HD stimulated with *Mtb*-Ag secreted the highest levels of IFN-γ. Together, our present findings *in vitro* are in line with our genetic association results, indicating that the A allele and AA genotype of the rs2275913 SNP would be a biomarker of resistance to tuberculosis disease in the Argentinean population.

When we analyzed the production of cytokines by the different rs2275913 carriers within the TB population, we observed that PBMC from AA TB produced the highest levels of IL-17A in cultures supernatants and that *Mtb*-Ag induced the most elevated number of T cells secreting this cytokine. These results emphasize the idea that the A allele is associated with a higher production of IL-17A^30^. However the levels of IL-17A detected were higher than those produced by HD, probably because tuberculosis disease exacerbates the secretion of this cytokine^22^. Moreover, we previously demonstrated that exogenous addition of recombinant IL-17A to cells in culture inhibited IFN-γ production against the pathogen^23^. Actually, in our present results we evidenced that AA TB displayed the lowest levels of IFN-γ and the highest amounts of IL-17A. These findings are in agreement with studies suggesting that in a microenvironment with elevated levels of IL-17A, for those subjects that carry a genotype that favors a higher synthesis of this cytokine, the situation might be detrimental, leading to a reduction of IFN-γ production, recruitment of excessive numbers of neutrophils and tissue damage^18^,^20^,^22^.

Considering the results described above, our second hypothesis was that TB carrying the AA genotype of the rs2275913 SNP, individuals that displayed the highest levels of IL-17A and the lowest amounts of IFN-γ, might be the TB most affected by tuberculosis disease. Previously, we showed that several immunological parameters evaluated *in vitro* allow to discriminate TB in High and Low Responder (HR, LR), where LR individuals display the most severe tuberculosis^39^. In the present work, we found that AA TB display the lowest levels of the immunological parameters measured (SLAM expression in CD3^+^ T cells, proliferation index and IFN-γ production) as compared to TB carrying the other genotypes. Furthermore, when we classified the TB population in HR and LR and we analyzed the distribution of allelic and genotypic frequencies of the rs2275913 SNP, we found that the AA genotype and the A allele are more frequent in LR TB. Therefore, the immunological parameters allow us to associate the A variant of the rs2275913 SNP with severe tuberculosis.

It has been demonstrated the existence of a correlation between immunological and clinical parameters in tuberculosis in Argentina^39^,^40^. Later, we found that the presence of higher proportions of CD4^+^ IFN-γ^+^ IL-17A^+^ lymphocytes was correlated directly with more extensive lung affectation and a higher number of pulmonary lesions, indicating disease severity^22^. We next asked whether there was any association between the rs2275913 SNP variants and clinical parameters measured in TB, as reported for other SNPs^45^,^46^,^47^. We did not find significant differences in the time of disease progression. We neither evidenced differences in the hematologic counts in peripheral blood between TB carrying the different genotypes. It is well established that IL-17A is linked to neutrophil influx at the site of infection^18^,^48^,^49^. We believe that studies in bronchoalveolar lavages would be required to elucidate if AA TB individuals, who secrete higher IL-17A levels, displayed increased neutrophils recruitment to the site of infection. On the other hand, we did find that the majority of AA TB carried the highest bacterial burden in sputum (BAAR) and the most severe pulmonary lesions. Then, these results support our theory of the association between the AA genotype and severe clinical parameters of the disease (i.e.: far advanced pulmonary lesions, high bacillary loads). Furthermore, our data are in line with other reports showing correlation of IL-17A production in TB with disease severity^22^ and with an elevated bacterial burden^24^.

In conclusion, we found that AA genotype is associated with resistance to active tuberculosis, a fact that comes from the case control study where we found that the frequency of AA individuals in TB population were statistically reduced in comparison to the frequency of AA individuals in HD population. Additionally, considering the widely demonstrated importance of Th1 responses against infection with *Mtb*, our *in vitro* studies showed that AA PBMC from HD individuals secreted higher levels of IFN-γ against *Mtb*-Ag in comparison to PBMC from GG subjects. Actually, when we measured and compared the proliferation index and the SLAM expression on five days *Mtb*-Ag stimulated PBMC from HD carrying the different rs2275913 variants (data not shown), we observed that both parameters are statistically increased in PBMC from AA HD in comparison with GG HD, reinforcing the idea that AA individuals display a stronger cellular immunity against *Mtb*. Taking together, all these results indicate that the AA genotype of the rs2275913 SNP would be a biomarker of resistance to tuberculosis disease in the Argentinean population. However, the fact that the AA genotype is associated with protection does not imply that the individuals carrying this genotype display full immunity to tuberculosis. Therefore, if the subjects get tuberculosis disease, and considering they carry a genotype that induce their cells to produce higher levels of IL-17A, that may expose these individuals to a worse disease outcome. We evidenced that *Mtb*-Ag stimulated PBMCs from AA TB secreted the highest levels of IL-17A, the lowest levels of IFN-γ, expressed the lowest levels of SLAM in CD3^+^ T cells and had the lowest proliferation index in comparison with GG TB, suggesting they display a weaker immunity against *Mtb*. In fact, we also found evidence that associated the AA genotype in TB with clinical parameters of disease severity. IL-17A/IFN-γ double positive CD4 cells would not be an immune mechanism associated to the shift we observed when an AA HD get tuberculosis, considering that AA HD and AA TB displayed the highest percentage of IL-17/IFN-γ double positive CD4 cells in *Mtb*-Ag stimulated PBMCs as compared to GG/GA HD or GG/GA TB respectively (Supplementary Figure S2). Our findings suggest that certain levels of IL-17A would be required to carry out the immune mechanisms necessary for the rapid elimination of the pathogen without interfering with the Th1 response^19^,^49^. However, when tuberculosis disease is already established, the levels of IL-17A are increased, and augmented secretion of this cytokine, as in AA TB patients, would be detrimental as a result of an exacerbated inflammation at the site of infection and an inhibition of the Th1 response^18^,^22^,^23^. Further immunological studies are under investigation to elucidate the fact that AA genotype is associated with tuberculosis resistance but is related to severity of tuberculosis if the individual gets the disease.

Taken together, we describe for the first time, the existence of an association between the A allele form the rs2275913 SNP of the IL-17A and resistance to tuberculosis disease in Argentina. This allele was found to be also associated with a higher IL-17A production in both HD and TB populations. However, in TB the production of IL-17A would be higher as compared to HD, since tuberculosis exacerbates its secretion^22^. Moreover, the fact of carrying a particular genotype that augments even more the synthesis of IL-17A would be harmful for tuberculosis outcome, given that we detected an association between AA genotype with clinical and immunological parameters of disease severity.

The identification of the rs2275913 AA genotype as a biomarker of tuberculosis protection and the role of the IL-17A in the immune-physiology of tuberculosis might contribute to design more effective vaccines and to identify risky sub-populations in Argentina. Furthermore, the association of the rs2275913 AA genotype with higher tuberculosis severity might collaborate in the implementation of new host-directed treatment strategies.

## Materials and Methods

### Samples

185 HIV-uninfected patients with active tuberculosis (TB) were diagnosed at the Dr. F. J. Muñiz and the Dr. E. Tornú Hospitals (Buenos Aires, Argentina), based on clinical and radiological data, together with the identification of acid-fast bacilli in sputum and/or isolation of *Mtb* in culture. Patients included in this study had received less than 1 week of anti-tuberculosis therapy. TB were classified as High Responder (HR) patients (individuals displaying high proliferative responses, IFN-γ production, and SLAM expression in CD3^+^ cells against *Mtb*-Ag) and Low Responder (LR) patients (individuals that exhibit low proliferative responses, IFN-γ secretion, and percentages of SLAM^+^ CD3^+^ cells), as previously described^39^. 207 healthy donors (HD) were recruited and included individuals who had received BCG vaccination at birth, were potentially exposed to *Mtb* (tuberculosis contacts), and lacked a history of tuberculosis. Moreover, family contacts that were negative for the QuantiFERON-TB Gold In-Tube test (QFT-GIT, Qiagen, USA) were also included in the HD group. Diagnosis of latent tuberculosis infection was assigned to any subject with QFT-GIT positive results and no clinical or radiological evidence of active TB. These individuals were excluded from the study. All participants provided a written, informed consent for the collection of samples and subsequent analysis. All the individuals participating in this study were over 18 years old. All methods were carried out in accordance with relevant guidelines and regulations. The protocols conducted in this work were approved by the Ethical Committee of the Dr. F. J. Muñiz and the Dr. E. Tornú Hospitals.

### Antigen

*In vitro* stimulation of cells throughout the study was performed with a cell lysate from the virulent *M. tuberculosis* H37Rv strain, prepared by probe sonication (*Mtb*-Ag), and obtained through BEI Resources, NIAID, NIH: Mycobacterium tuberculosis, Strain H37Rv, Whole cell lysate, NR-14822 (Bethesda, MD, USA).

### DNA Extraction, SNP Primers Design and Genotyping

Genomic DNA was extracted from whole blood samples using the Quick-gDNA™ Blood MiniPrep (Zymo Reasearch, California, USA) according to the manufacturer’s instructions. DNA purity and final concentrations were determined spectrophotometrically. Amplification refractory mutation system-polymerase chain reaction (ARMS-PCR) was used for the rs2275913 SNP genotyping. The ARMS-PCR is based on allele specific amplification of desired fragment using primers corresponding to each allelic variant^50^. Primer sequences were designed by the BeaconDesigner 7.2 software (Premier Biosoft International, Ltd., Palo Alto, CA, USA). The sequences of the primers used are: Allele A specific forward 5′ATGGTGTTAATCTCATCTGGTGGG3′, Allele G specific forward 5′ATGGTGTTAATCTCATCTGGTGGC3′, Common reverse 5′ATGCCCACGGTCCAGAAATAC3′. As an internal control, Human Growth Hormone (HGH) gene primers (Forward 5′GCCTTCCCAACCATTCCCTTA3′, Reverse 5′ TCACGGATTTCTGTTGTGTTTC 3′) were included in every PCR mix to verify successful amplification. The amplification was performed in a Multigene Gradient thermal cycler (LabNet International, NJ, USA). The conditions included initial denaturation (94 °C for 5 min) following a 35 time cycles of denaturation at 94 °C for 30 s, annealing at 58 °C for 50 s and extension at 72 °C for 45 s each cycle; and final extension at 72 °C for 5 min. rs2275913 genotypes were assessed from the presence/absence of PCR amplicon (312 bp), corresponding to the specific allele (A/G) on 1.5% agarose gel stained with SYBR Green. All genotypes of the rs2275913 SNP were confirmed by direct sequencing of the amplified IL-17A gene fragment by Sanger method (ABI 3130xl GeneticAnalyzer, Applied Biosystems, USA), and a 100% concordance was obtained among the results obtained from ARMS-PCR and DNA sequencing (Supplementary Figure S1).

### Cell Preparation and Reagents

Plasma samples were collected by blood separation with centrifugation at 2,500rpm for 10 min, and the samples were stored at −80 °C until IL-17A (eBioscience, CA, USA) and IFN-γ (BioLegend, CA, USA) determination by ELISA was performed. Peripheral blood mononuclear cells (PBMC) were isolated by centrifugation over Ficoll-Hypaque (Amersham Biosciences, NJ, USA) and cultured (1 × 10^6^ cells/mL), with or without *Mtb*-Ag (10 μg/mL) with RPMI 1640 medium (Gibco, MD, USA) supplemented with 1% L-glutamine, 1% penicillin/streptomycin, and 10% human serum (Sigma-Aldrich, MO, USA) during 48 hours or five days. Then, IL-17A and IFN-γ expression was determined by ELISA and flow cytometry.

### Flow Cytometry

PBMC were stimulated with *Mtb*-Ag for five days and incubated with monensin (1 μl/ml; Sigma-Aldrich, MO, USA) for the last five hours of culture. Cells were then stained with specific fluorophore-marked antibodies against CD3 (FITC, UCHT1, BioLegend), CD4 (FITC, RPA-T4, BioLegend), SLAM (PE, A12, BD Pharmingen). Intracellular staining was performed to determine IL-17A (PECy7, eBio64DEC17, eBioscience) and IFN-γ (APC, 4S.B3, eBioscience) expression. For intracellular cytokine staining, permeabilization buffer containing 0.5% saponin (Sigma-Aldrich, MO, USA) and 10% fetal bovine serum (Gibco, MD, USA) in PBS was used. Negative control samples were incubated with irrelevant isotype-matched mAb in parallel with experimental samples, which were analyzed on a FACSAria II flow cytometer (BD Biosciences).

### Proliferation Index

PBMC were stimulated with *Mtb*-Ag for five days and cells were pulsed with [^3^H]TdR (1 μCi/well) and harvested 16 h later. [^3^H]TdR incorporation was measured in a liquid scintillation counter. Proliferation index for each individual was calculated as cpm after *Mtb*-Ag stimulation/cpm after culturing with medium.

### Statistical Analysis

The genotype and allele frequencies were obtained by direct counting. Hardy–Weinberg (HW) equilibrium was tested between cases and controls separately (Chi-Square goodness-of-fit test). Comparisons of the distributions of the allele and genotype frequencies between case and control were performed using the Chi-Square test for homogeneity. An *a priori* sample size estimation with an initial population of 46 HD and 46 TB was performed. A sample of at least 116 individuals in each population was estimated to get a test power of 0,8. The level of association between the rs2275913 genotypes and the case/control condition was estimated as an odds ratio (OR) with a 95% confidence interval (CI)^31^. Additionally, OR calculation was also performed by logistic regression with adjustment for sex, ethnicity and age. The quantitative data were expressed as mean ± standard error of the mean (SEM), and the Mann–Whitney U test or the Kruskal-Whallis (ANNOVA) test for unpaired and non-parametric samples was used to analyze differences between groups. For categorical variables, the Chi-Square test for homogeneity was performed to compare proportions of subjects between groups and the Chi-Square goodness-of-fit test was used to evaluate deviations from Hardy-Weinberg equilibrium. All statistical analysis were performed using GraphPad Prism v6.0 (GraphPad Software, CA, USA). *P* values of <0.05 were considered statistically significant.

## Additional Information

**How to cite this article**: Rolandelli, A. *et al*. The IL-17A rs2275913 single nucleotide polymorphism is associated with protection to tuberculosis but related to higher disease severity in Argentina. *Sci. Rep.* **7**, 40666; doi: 10.1038/srep40666 (2017).

**Publisher's note:** Springer Nature remains neutral with regard to jurisdictional claims in published maps and institutional affiliations.

## Supplementary Material

Supplementary Information

## Figures and Tables

**Table 1:** Demographic characteristics of healthy donors and tuberculosis patients populations.

		HD	TB	χ^2^ or U value	*P value*
N		207	185		
Age (years)		33.8 ± 8.7	33.5 ± 14.5	5885^a^	0.073^a^
Ethnicity	Caucasian	70.90%	62.00%	2.59^b^	0.107^b^
	American Indian	29.10%	38.00%		
Sex	Male	39.13%	78.37%	54.49^b^	<0.001^b^
	Female	60.87%	21.62%		

Categorical variables are expressed in percentages. Age value is expressed as mean ± SEM. ^a^*P* values were calculated by the Mann-Whitney U test for unpaired samples. ^b^*P* values were calculated by Chi-Square test for categorical variables. HD: healthy donors; TB: tuberculosis patients.

**Table 2:** Association between clinical parameters and the rs2275913 SNP genotypic variants during active tuberculosis.

Tuberculosis patients	rs2275913 genotype:			χ^2^ or F value	*P value*
	GG	GA	AA		
Hematologic Studies (n = 86)					
Leukocytes (cells/ml)	10230 (±460.9)	9350 (±711.4)	14600 (±3530)	4273^a^	0.118^a^
Lymphocytes (cells/ml)	1613 (±88.52)	1381 (±143.5)	1649 (±164.1)	3873^a^	0.144^a^
Monocytes (cells/ml)	920 (±49.53)	776.7 (±85.38)	1189 (±322)	4660^a^	0.097^a^
Neutrophils (cells/ml)	7056 (±368.8)	6340 (±802.7)	4550 (±1274)	3956^a^	0.138^a^
AFB in sputum smear (n = 138)					
BAAR- or BAAR+	64 (70%)	26 (72%)	3 (30%)	6.942^b^	0.031^b^
BAAR++ or BAAR+++	28 (30%)	10 (28%)	7 (70%)		
Radiological Lesions (n = 110)					
Mild or Moderate	39 (57%)	9 (29%)	4 (40%)	4.282^b^	0.038^b^
Severe		22 (71%)	6 (60%)		
Months of disease progression (n = 89)	3.18 (±0.39)	2.98 (±0.38)	3.18 (±0.46)	0.981^a^	0.612^a^

Hematologic studies representing the leukocyte, lymphocyte, monocyte and neutrophil counts in peripheral blood are shown. Acid-Fast Bacilli (AFB) in sputum smear represent: BAAR-, 0 bacilli count; BAAR+, 1–9 bacilli/100 fields; BAAR++, 1–9 bacilli/10 fields; BAAR+++, 1–9 bacilli/field. Radiological lesions: mild corresponds to patients with a single lobe involved and without visible cavities; moderate relates to patients presenting unilateral involvement of two or more lobes with cavities, if present, reaching a total diameter no greater than 4 cm; severe corresponds to bilateral disease with massive affectation and multiple cavities. Clinical symptoms analyzed in tuberculosis patients previous to hospital admission to establish the time (months) of disease progression were: weight loss, night sweats, symptoms of malaise or weakness, persistent fever, presence of cough, history of shortness of breath, and hemoptysis. Continuous data are expressed as Mean ± SEM, and categorical data are expressed as number (percentages of genotype). ^a^*P* values were calculated by the Kruskal-Wallis (ANOVA) test for unpaired and non-parametric samples. ^b^*P* values were calculated by the Chi-square test for categorical variables.
