# Supplementary material for: The IL-17A rs2275913 single nucleotide polymorphism is associated with protection to tuberculosis but related to higher disease severity in Argentina
Source: Sci Rep. 2017 Jan 18;7:40666. doi: 10.1038/srep40666 (PMC5241634; doi:10.1038/srep40666)
Supplement: Supplementary Information [file srep40666-s1.pdf]

Supplementary information

The IL-17A rs2275913 single nucleotide polymorphism is associated with protection to tuberculosis but related to higher disease severity in Argentina

Rolandelli, A.[1,2]; Hernández Del Pino, R. E.[1,3]; Pellegrini, J. M.[1,2]; Tateosian, N. L.[1,2]; Amiano, N.O.[1,2]; de la Barrera S[4]; Casco, N.[5]; Gutiérrez, M.[6]; Palmero, D. J.[5]; García, V. E*[1,2]

[1] Departamento de Química Biológica. Facultad de Ciencias Exactas y Naturales. UBA, Intendente Güiraldes 2160, Pabellón II, 4°piso, Ciudad Universitaria (C1428EGA), Buenos Aires, Argentina.

[2] Instituto de Química Biológica, Facultad de Ciencias Exactas y Naturales (IQUIBICEN), UBA (Universidad de Buenos Aires)-CONICET, Intendente Güiraldes 2160, Pabellón II, 4°piso, Ciudad Universitaria (C1428EGA), Buenos Aires, Argentina.

[3] Centro de Investigaciones y Transferencia del Noroeste de Buenos Aires (CIT NOBA), CONICET. Newbery 261, Junín (6000), Buenos Aires, Argentina.

[4] Instituto de Medicina Experimental-CONICET-Academia Nacional de Medicina. Pacheco de Melo 3081 (CP1425), Buenos Aires, Argentina.

[5] División Tisioneumonología Hospital F.J. Muñiz, Uspallata 2272, (C1282AEN) Buenos Aires, Argentina.

[6] Sección Bacteriología de la Tuberculosis, Hospital General de Agudos “Dr. E. Tornu”, Combatientes de Malvinas 3002, (C1427ARN) Buenos Aires, Argentina

*vgarcia@qb.fcen.uba.ar

Supplementary Table S1.

		HD (N=207)			TB (N=185)		
**rs2275913 genotypes**		GG	GA	AA	GG	GA	AA
**Sex**	Male	40	31	10	98	41	6
	Female	68	45	13	26	10	4
P value		0.79			0.34		

Genotypic frequencies of the IL-17A rs2275913 SNP in HD and TB populations stratified by sex. *P* values were calculated by the Chi-Square test for categorical variables. HD: healthy donors; TB: tuberculosis patients.

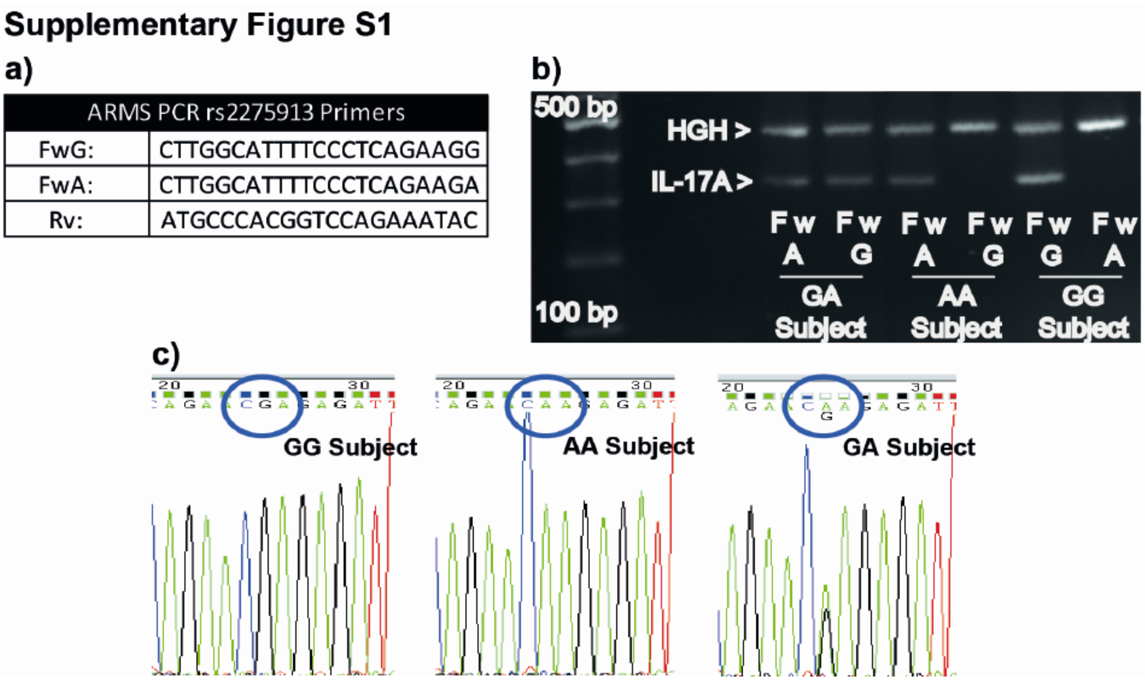

Supplementary Figure S1. IL-17A rs2275913 SNP genotyping by ARMS-PCR method. (a) Primer sequences (two Forward primers and one common Reverse primer) designed to specifically amplify a 317 pb amplicon that discriminates both alleles of the rs2275913 SNP. **(b)** Image of an agarose gel displaying the PCR products obtained from three genotypically different individuals for the SNP under study is shown. PCR positive control: Human Growth Hormone (HGH) gene fragment (440 bp). rs2275913 genotypes were assessed from the presence/absence of PCR amplicon corresponding to the specific allele (A or G). **(c)** DNA sequencing of the amplicons obtained from three genotypically different individuals. Primers specificity of the rs2275913 SNP were confirmed by direct sequencing of the amplified IL-17A gene fragment by Sanger method and a 100% concordance was obtained among the results obtained from ARMS-PCR and DNA sequencing.

Supplementary Figure S2

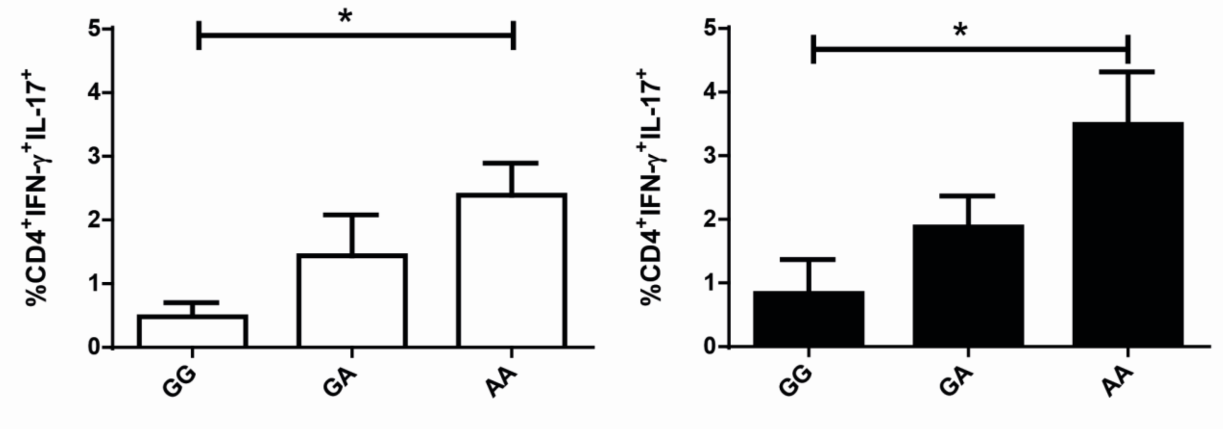

Supplementary Figure S2. Percentage of IFN-γ$^+$IL-17A$^+$CD4$^+$T cells in *Mtb*-Ag stimulated PBMCs from HD and TB carrying the genotypic variants of the rs2275913 SNP. PBMCs from HD (n=15, white bars, left panel) and TB (n=16, black bars, right panel) carrying the different genotypes of the rs2275913 SNP were stimulated for five days with *Mtb*-Ag, and IFN-γ$^+$IL-17A$^+$CD4$^+$T cells percentage was determined by Flow Cytometry. The percentages represent an increase in the number of cytokine-positive CD4$^+$T cells in response to *Mtb*-Ag stimulation. IL-17A and IFN-γ expression was determined gating on lymphocytes by light scatter first, and then gating on CD4$^+$T cells. Bars represent the Mean ± SEM. P values were calculated by the Kruskal-Wallis (ANOVA) test for unpaired and non-parametric samples. *$P<0.05$.
